# Lectures on Auto-Intoxication in Disease, or Self-Poisoning of the Individual

**Published:** 1906-02

**Authors:** 


Lectures on Auto-Intoxication in Disease, or Self-Poisoning of the Individual. By. Ch. Bouchard, Professor of Pathology and Therapeutics; Member of the Academy of Medicine and Physician to the Hospitals, Paris. Translated, with a preface and new chapters added, by Thomas Oliver, M. A., M. A., M. D., F. R. C. P., Professor of Physiology, University of Durham; Physician to the Royal Infirmary, New Castle-Upon-Tyne; Formerly Examiner in Medicine, Royal College of Physicians, London. Second revised edition. Crown octavo, 342 pages, extra cloth. Price, $2, net. F. A. Davis Company, Publishers, 1914-16 Cherry street, Philadelphia.

This book treats of subjects of everyday interest to the physician. Many of the facts therein alluded to can not be ignored. Putrefactive processes in the intestinal canal and the development of physiological and pathological alkaloids play an important part in many disease processes until lately unknown or not understood.

These lectures are an inquiry into the operation of poisons introduced from without or generated within the body of man, and the part they play in health and disease. No subject commands a greater interest; none demands more serious study. Fatal cases of ptomaine poisoning from eating canned goods or cold-storage meats and fish and game, are alarmingly frequent. D.

